# Self-perception of swallowing, vocal symptoms, and the possibility of dysphagia occurrence in patients under palliative care

**DOI:** 10.1590/2317-1782/e20250208en

**Published:** 2026-05-08

**Authors:** Sophia Eustáquio Rodrigues, Gabriel Trevizani, Michelle Guimarães, Elma Heitmann

**Affiliations:** 1 Departamento de Fonoaudiologia, Universidade Federal do Espírito Santo – UFES - Vitória (ES), Brasil.; 2 Programa de Pós-graduação em Ciências da Saúde e Comunicação Humana, Universidade Estadual Paulista "Júlio de Mesquita Filho" – Unesp - Marília (SP), Brasil.

**Keywords:** Deglutition, Deglutition Disorders, Voice, Palliative Care, Mass Screening, Speech, Language and Hearing Sciences

## Abstract

**Purpose:**

To identify, compare, and correlate self-perception of swallowing, vocal symptoms, and the possibility of dysphagia in patients under Palliative Care (PC).

**Methods:**

Observational, cross-sectional, and analytical study, approved by the Research Ethics Committee (nº 6.039.141), with a convenience sample collected between April/2023 and May/2024. Clinical data were obtained from medical records. Included were hospitalized patients in the PC unit, of both sexes, aged over 18 years. Exclusion criteria were lack of clinical conditions and/or willingness to participate. After signing the informed consent form, patients completed the EAT-10, VoiSS, and RaDI-H protocols.

**Results:**

A total of 50 patients participated, with a prevalence of males (58%), over 60 years of age (64%), cancer diagnosis (66%), and oral feeding route (74%). Sarcopenia (46%) and dyspnea (52%) were observed. Negative self-perception of swallowing was found in 80% of patients. On the total VoiSS score, 58% scored above the cutoff, with the greatest impact on the limitation domain (44%). Dysphagia was possible in 62% of patients. There was a significant difference between patients with and without sarcopenia regarding self-perception of swallowing, limitation domain, total VoiSS score, and possibility of dysphagia. Significant differences were also found between patients with and without dyspnea reports in all three protocols, except for the emotional domain of the VoiSS. Positive correlations were observed among the protocols.

**Conclusion:**

In these PC patients, there was negative self-perception of swallowing, self-reported vocal symptoms, and possibility of dysphagia, with positive correlation among the three protocols. Significant differences were found in self-perception of swallowing, vocal symptoms, and possibility of dysphagia, with greater impact in patients with sarcopenia and dyspnea.

## INTRODUCTION

Palliative Care (PC) is an approach aimed at promoting quality of life for patients and their families through the early assessment and management of distressing physical, social, emotional, and spiritual symptoms in the context of life-threatening illnesses^(1).^ Care is delivered by a multidisciplinary team throughout the stages of diagnosis, disease progression, end-of-life, and bereavement, providing support to both patients and families according to their preferences and needs^(1,2)^.

Within this context, speech-language pathologists play a fundamental role in the management of swallowing and communication, guided by the bioethical principles of autonomy, beneficence, non-maleficence, and justice. Their practice involves the assessment and management of dysphagia, the adaptation of communication strategies, and support for shared decision-making, while respecting patients’ preferences and wishes. The ultimate goal is to alleviate suffering, preserve dignity, and maintain quality of life until the end of life^(3)^.

In Brazil, the estimated number of patients requiring PC in 2000 was 662,065, and projections indicate a minimum of 1,166,279 individuals by 2040, demonstrating the steadily increasing demand for PC services^(2)^.

Patients receiving PC frequently present with dysphagia and dysphonia due to neurodegenerative or neoplastic diseases that may alter the physiology of swallowing and phonation^(4)^. Swallowing-related complaints are common in this population^(5,6)^, and approximately 70% of patients exhibit dysphagia for at least one food consistency^(5)^.

Another study^(6)^, conducted in a PC service, revealed that 56% of patients presented with dysphonia, with a significant reduction in maximum phonation time, as well as alterations in speech articulatory organs and the presence of dysarthrophonia. These findings underscore the need for a comprehensive assessment of vocal and speech motor function in patients receiving PC, as such impairments may significantly affect communication and quality of life^(6)^. Early identification of these problems is crucial for the development of intervention strategies aimed at improving vocal function and communication capacity in this population.

Screening for the risk of dysphagia represents a simple and effective strategy to minimize its impact through early intervention, while also contributing to patients’ quality of life^(7)^. In a study conducted at a university hospital with a sample of 909 patients, the risk of dysphagia was identified in 10.5% of individuals; among those at risk, 4.2% presented with dysphonia, which is considered a risk factor for the development of dysphagia^(7)^.

Given the prevalence of swallowing and voice-related symptoms in patients receiving PC, it is essential to investigate self-perceived swallowing function, vocal symptoms, and the likelihood of dysphagia. Self-assessment may be performed using specific validated self-report instruments as a strategy to reinforce early speech-language pathology intervention. In doing so, the primary objective of PC is fulfilled: to promote quality of life and to support informed clinical decision-making. Therefore, this study aimed to identify, compare, and correlate self-perceived swallowing function, vocal symptoms, and the likelihood of dysphagia in patients receiving PC.

## METHODS

This was an observational, cross-sectional, and analytical study approved by the Institutional Research Ethics Committee under protocol number 6,039,141. All participants provided written informed consent before enrollment.

Data collection was conducted using a convenience sample between April 2023 and May 2024. Participants were hospitalized patients receiving care in the PC unit of a university hospital, with different underlying diagnoses. Patients were categorized according to the predominance of palliative care as follows: complementary care, predominant care, exclusive (total) care, and end-of-life/terminal phase^(8,9)^. Individuals aged 18 years or older were eligible, with no restriction regarding sex. Patients without clinical conditions and/or willingness to complete the assessment protocols were excluded.

Clinical and sociodemographic data were obtained through medical record review. To screen for the risk of sarcopenia, the SARC-F questionnaire was administered^(10)^. Participants completed three self-report instruments in a single assessment session:

1. The Eating Assessment Tool (EAT-10) is a self-perception instrument designed to assess swallowing function in patients with various underlying conditions. It consists of 10 items scored on a 5-point Likert scale ranging from 0 (no problem) to 4 (severe problem). The instrument provides information on functional limitations, emotional impact, and physical symptoms related to swallowing impairment. A total score ≥3 indicates negative self-perception of swallowing and the need for formal speech-language pathology assessment^(11,12)^.
2. The Voice Symptom Scale (VoiSS) is considered one of the most rigorous and psychometrically robust instruments for vocal self-assessment^(13)^. It evaluates the perception of vocal symptoms and their impact on voice through 30 items divided into three domains: limitation (15 items), emotional (8 items), and physical (7 items). Items are rated according to symptom frequency: never (0), rarely (1), sometimes (2), almost always (3), and always (4). Total scores range from 0 to 120, with higher scores indicating worse self-perceived vocal symptoms. Cutoff scores for differentiating vocally healthy individuals from dysphonic patients are: 16 points for the total score, 11.5 for the limitation domain, 6.5 for the physical domain, and 1.5 for the emotional domain^(13)^.
3. The *Rastreamento de Disfagia Orofaríngea em Idosos no ambiente hospitalar* (RaDI-H) is a self-assessment tool designed to screen for dysphagia in hospitalized older adults. It comprises nine straightforward questions aimed at identifying swallowing-related symptoms and complaints^(14,15)^. Response options include “no,” “yes,” or “do not know.” In cases of affirmative responses, symptom frequency is further specified as “sometimes” or “always.” Scores range from 0 to 2 per item (“no” = 0; “yes” = 2; “sometimes” = 1; “always” = 2; “do not know” = 2)^(14,15)^. Total scores range from 0 to 18 points. A cutoff score of ≥4 indicates a positive screening result and the need for referral for diagnostic evaluation^(14,15)^.

Participants who demonstrated negative self-perception of swallowing, presence of vocal symptoms, and/or positive screening for dysphagia were referred to the Speech-Language Pathology team for formal assessment and follow-up.

For statistical analysis, Microsoft Excel and Jamovi software (version 2.0) were used. The Mann–Whitney U test was applied to compare EAT-10, VoiSS, and RaDI-H scores. Spearman’s rank correlation coefficient was used to assess correlations among instrument scores. A significance level of 5% (p ≤ 0.05) was adopted. Correlation coefficients were classified as follows: <0.3 (weak), ≥0.3 to <0.6 (moderate), ≥0.6 to <0.9 (strong), ≥0.9 to <1.0 (very strong), and r = 1.0 (perfect)^(16)^.

## RESULTS

A total of 50 patients were included, of whom 29 (58%) were male. Most participants were older than 60 years (64%), with a mean age of 67 years (±13.6). Regarding the underlying diagnosis, 33 patients (66%) had cancer. Oral feeding was the primary route of nutrition for 37 patients (74%). Risk of sarcopenia was identified in 23 patients (46%), and 26 (52%) self-reported dyspnea.

Negative self-perception of swallowing was observed in 40 patients (80%). Concerning vocal symptoms, 29 patients (58%) had total VoiSS scores above the cutoff value. In the limitation domain, 22 patients (44%) scored above the cutoff, followed by the physical (n = 16; 32%) and emotional (n = 11; 22%) domains. Positive screening for dysphagia (RaDI-H) was observed in 31 patients (62%).

The mean EAT-10 score was 10.5 (SD ±10.4), with a median of 7, exceeding the established cutoff. For the VoiSS, the limitation domain showed a mean score of 14.5 (SD ±12) and a median of 12, both above the cutoff. The emotional domain had a mean of 1.24 (SD ±3) and a median of 0, below the cutoff, while the physical domain presented a mean of 6 (SD ±5) and a median of 5, also below the cutoff. The mean total VoiSS score was 21.6 (SD ±19), with a median of 19.5, exceeding the cutoff value. The mean RaDI-H score was 5.44 (SD ±3.8), with a median of 4, also above the cutoff (Table 1).

**Table 1:** Descriptive statistics of EAT-10, VoiSS, and RaDI-H scores in patients receiving Palliative Care (n = 50)

Variables	EAT 10	VoiSS Limitation	VoiSS Emotional	VoiSS Physical	VoiSS Total	RaDI-H
Mean (± SD)	10.5 (±10.4)	14.5 (± 12)	1.24 (± 3)	6 (± 5)	21.6 (± 19)	5.44 (± 3.8)
Median	7	12	0	5	19.5	4

**Caption:** SD = standard deviation; EAT-10 = Eating Assessment Tool; VoiSS = Voice Symptom Scale; RaDI-H = *Rastreamento de Disfagia Orofaríngea em Idosos em Ambiente Hospitalar*

Statistically significant differences were observed between individuals with and without sarcopenia regarding self-perceived swallowing (p = 0.005), the VoiSS limitation domain (p = 0.041), total VoiSS score (p = 0.044), and risk of dysphagia (p = 0.007). No significant differences were found for the VoiSS physical (p = 0.102) or emotional (p = 0.765) domains, although patients with sarcopenia exhibited higher mean scores (Table 2).

**Table 2:** Comparison of EAT-10, VoiSS, and RaDI-H scores according to clinical characteristics of patients receiving Palliative Care (n = 50)

Variables		Sarcopenia			Dyspnea		
		Yes	No	p-value	Yes	No	p-value
EAT-10		12	8.7	0.005*	15.4	6.2	0.001*
VoiSS	Limitation	19,8	8,6	0.041*	18.6	10.9	0.001*
	Emotional	1.5	0.9	0.765	1.2	1.2	0.233
	Physical	8.4	3.3	0.102	6.9	5	0.001*
	Total	29	12.9	0.044*	26.8	17	0.001*
RaDI-H		7	3.6	0.007*	6.9	4	0.001*

* Statistically significant difference at p ≤ 0.05;

**Caption:** EAT-10 = Eating Assessment Tool; VoiSS = Voice Symptom Scale; RaDI-H = *Rastreamento de Disfagia Orofaríngea em Idosos em Ambientes Hospitalares*

Significant differences were also observed between patients with and without self-reported dyspnea (p = 0.001) across all instruments, except for the VoiSS emotional domain, which showed identical mean values between groups (p = 0.233) (Table 2).

Strong positive correlations were identified between EAT-10 and RaDI-H (r = 0.664), and between EAT-10 and the VoiSS physical domain (r = 0.653). Moderate positive correlations were found between EAT-10 and the VoiSS limitation domain (r = 0.492), as well as the total VoiSS score (r = 0.556). The RaDI-H demonstrated strong positive correlations with the VoiSS limitation (r = 0.654), physical (r = 0.644), and total scores (r = 0.689). Additionally, weak positive correlations were observed between EAT-10 and the VoiSS emotional domain (r = 0.010), and between RaDI-H and the VoiSS emotional domain (r = 0.246) (Table 3).

**Table 3:** Correlation among EAT-10, VoiSS, and RaDI-H scores in patients receiving Palliative Care (n = 50)

Variables	EAT 10	RaDI-H
RaDI-H	0.664**	
VoiSS Limitation	0.492**	0.654**
VoiSS Emotional	0.010**	0.246**
VoiSS Physical	0.653**	0.644**
VoiSS Total	0.556**	0.689**

** Spearman’s rho correlation coefficient;

**Caption:** EAT-10 = Eating Assessment Tool; VoiSS = Voice Symptom Scale; RaDI-H = *Rastreamento de Disfagia Orofaríngea em Idosos em Ambientes Hospitalares*

## DISCUSSION

The clinical profile of patients receiving PC varies across studies^(17)^, although a predominance of male patients older than 60 years has frequently been reported, consistent with the findings of the present study. This distribution may be associated with lower levels of self-care among men compared to women and a greater propensity for harmful lifestyle behaviors, such as the use of illicit substances, alcohol, and tobacco^(17)^, which may contribute to increased comorbidities and more severe health complications^(17)^.

With respect to age, older patients are more commonly affected by degenerative diseases and complications resulting from the natural physiological decline associated with aging, thereby intensifying the need for PC^(17)^. A 2019 study^(17)^ reported that 54% of patients were male, similar to our findings, although the predominant age range was 41–50 years. In contrast, another study^(18)^ identified a higher prevalence of female patients over 85 years old. These discrepancies suggest that patient profiles may vary depending on contextual and population-specific characteristics.

The high prevalence of cancer in PC settings is attributable both to its elevated incidence and to the substantial symptom burden associated with the disease and its treatment-related sequelae^(19,20)^. Studies have reported a high prevalence of negative self-perception of swallowing in patients with advanced cancer, ranging from 58% to 77%^(19,20)^, frequently accompanied by vocal symptoms and risk of dysphagia^(21)^. These findings underscore the importance of assessing swallowing self-perception, the impact of vocal symptoms, and screening for dysphagia in this population.

Oral feeding offers clinical and functional benefits, including reduced length of hospital stay, lower incidence of clinical complications, and decreased caregiver burden. It also encompasses sociocultural and emotional dimensions that are highly relevant to quality of life^(22,23)^. Na In the present study, oral feeding was the predominant route of nutrition. This route is considered less invasive, associated with better quality of life, and is often preferred by families when compared with alternative feeding methods^(23)^. However, our findings, in line with previous reports^(22-24)^, demonstrate that exclusive oral feeding does not preclude the presence of dysphagia or negative self-perception of swallowing.

Most patients (80%) reported negative self-perception of swallowing. The literature supports these findings, indicating that impaired swallowing perception is associated with reduced quality of life in PC populations^(6,25)^. Individuals in the advanced stages of life are particularly vulnerable to alterations in swallowing safety and efficiency due to multiple factors, including the effects of the underlying disease and its treatment, structural changes, sensory and sensorimotor impairments, fatigue, medication use, and cognitive decline^(25)^. This multifactorial burden contributes to functional deterioration of swallowing and directly influences patients’ subjective perception of their swallowing ability^(6,25)^.

Regarding vocal self-perception, 58% of patients presented total scores comparable to those observed in dysphonic individuals^(13)^, reflecting a negative perception of vocal function. Despite the relevance of this finding, no studies were identified that specifically investigate vocal self-perception in PC populations, highlighting an important gap in the literature.

According to Gabriel et al.^(6)^ dysarthrophonia and reduced maximum phonation time are among the most common vocal alterations observed in patients receiving PC. These alterations may contribute to negative vocal self-perception in this population.

The majority of participants (62%) screened positive for risk of dysphagia, reinforcing the relevance of speech-language pathology screening in PC settings. One study^(4)^ reported that approximately 30% of patients in PC described symptoms suggestive of penetration and/or aspiration during screening, with xerostomia being the most frequent complaint. Another investigation^(26)^ identified cough and choking as prevalent symptoms. Dysphagia has also been associated with increased disease burden, potentially leading to the emergence of new symptoms and negatively impacting quality of life^(26)^.

Statistically significant differences were observed between individuals with and without sarcopenia in terms of swallowing self-perception, the VoiSS limitation domain, total VoiSS score, and risk of dysphagia. These findings may be related to impairment of skeletal musculature involved in swallowing and voice production secondary to sarcopenia^(24)^. Loss of muscle strength and coordination may result in negative swallowing self-perception, increased dysphagia risk, and heightened perception of vocal symptoms^(24)^.

Muscle degradation in PC patients may result from malnutrition, physical inactivity, and chronic inflammation associated with advanced disease^(24)^, in addition to hormonal changes and the use of medications such as chemotherapeutic agents and corticosteroids^(24)^. Conditions such as cachexia may further accelerate the loss of muscle mass and function, exacerbating frailty^(24)^. The literature reports a higher prevalence of sarcopenia among oncology patients and males, a profile also identified in this study. In PC, sarcopenia has been associated with shorter survival^(24)^.

Patients who self-reported dyspnea demonstrated higher scores across most applied instruments, reflecting negative self-perception of swallowing, presence of vocal symptoms, and increased risk of dysphagia. This may be explained by impaired coordination between respiration and swallowing, increasing the likelihood of penetration and/or aspiration^(26)^.

Reduced subglottic pressure, glottic impairment, and the interaction between respiratory and muscular symptoms may contribute to the development of vocal symptoms in PC patients^(26)^. The coexistence of sarcopenia, fatigue, dyspnea, and respiratory alterations compromises pneumophonoarticulatory coordination, potentially resulting in compensatory phonatory effort and laryngeal overload, even if not voluntarily exaggerated^(6,24,25,27,28)^. These functional and structural laryngeal changes directly affect vocal quality and respiratory efficiency^(6,25)^. In this context, the interaction between respiratory and laryngeal systems becomes critical, as dysregulation may impair vocal quality and swallowing function, exacerbating symptoms such as hoarseness, cough, vocal fatigue, and swallowing difficulties, while increasing the risk of aspiration and related complications. Understanding this interplay is essential for effective therapeutic management in this population.

The positive correlations observed between swallowing self-perception, vocal symptoms, and dysphagia risk may be explained by the anatomical and muscular overlap of structures involved in these functions, particularly the larynx and pharynx^(6)^. Structural and neuromuscular alterations commonly observed in degenerative conditions may simultaneously compromise swallowing and voice, leading patients to perceive difficulties in both domains^(6)^. Negative swallowing self-perception and vocal symptoms often reflect alterations also identified during dysphagia screening, reinforcing the interrelationship among these variables in the diagnostic process^(25)^.

This study has limitations. Data were collected in a single hospital that is not a referral center for palliative care, and the sample size was relatively small, limiting generalizability. Additionally, low patient turnover and the presence of individuals in the active dying process or with delirium hindered full protocol administration. Future research should consider multicenter designs involving different PC services to enhance sample representativeness and allow broader generalization of findings. Including hospitals of varying sizes and specialties may also provide a more comprehensive understanding of the conditions and needs of this population.

## CONCLUSION

Patients hospitalized in the palliative care unit in this study demonstrated negative self-perception of swallowing, self-reported vocal symptoms, and a high likelihood of dysphagia. Statistically significant differences were observed in swallowing self-perception, presence of vocal symptoms, and risk of dysphagia, particularly among patients with sarcopenia and dyspnea. Furthermore, significant positive correlations were identified among the three assessed instruments, highlighting the interrelationship between swallowing impairment, vocal symptoms, and dysphagia risk in this population.
