# Supplementary material for: Self-perception of swallowing, vocal symptoms, and the possibility of dysphagia occurrence in patients under palliative care
Source: Codas. 2026 May 8;38(3):e20250208. doi: 10.1590/2317-1782/e20250208en (PMC13193196; doi:10.1590/2317-1782/e20250208en)
Supplement: Translation

# Translation: Autopercepção da deglutição, de sintomas vocais e da possibilidade de ocorrência da disfagia nos pacientes em cuidados paliativos

**DOI:** 10.1590/2317-1782/e20250208pt

**Authors:** Sophia Eustáquio Rodrigues, Gabriel Trevizani, Michelle Guimarães, Elma Heitmann

**Affiliations:** 1 Departamento de Fonoaudiologia, Universidade Federal do Espírito Santo – UFES - Vitória (ES), Brasil.; 2 Programa de Pós-graduação em Ciências da Saúde e Comunicação Humana, Universidade Estadual Paulista "Júlio de Mesquita Filho" – Unesp - Marília (SP), Brasil.

**Keywords:** Deglutição, Transtornos de Deglutição, Voz, Cuidados Paliativos, Programas de Rastreamento, Fonoaudiologia

## Abstract

**Objetivo:**

Identificar, comparar e correlacionar a autopercepção da deglutição, os sintomas vocais e a possibilidade de disfagia nos pacientes em Cuidados Paliativos (CP).

**Método:**

Estudo observacional, transversal e analítico, aprovado pelo CEP (nº 6.039.141), com amostra por conveniência, entre abril/2023 e maio/2024 e dados clínicos coletados dos prontuários. Foram incluídos pacientes internados, no setor de CP, independente de sexo e maiores de 18 anos, e excluídos pacientes sem condições clínicas e/ou interesse de participar. Após a assinatura do termo de consentimento, responderam aos protocolos EAT-10, ESV e RaDI-H.

**Resultados:**

Participaram 50 pacientes com prevalência do sexo masculino (58%), acima de 60 anos (64%), diagnóstico de câncer (66%) e via oral de alimentação (74%). Observou-se dados de sarcopenia (46%) e dispneia (52%). Houve 80% de autopercepção negativa da deglutição. No escore total da ESV, 58% pontuaram acima da nota de corte, com maior impacto no domínio limitação (44%). Houve possibilidade de disfagia em 62% dos pacientes, diferença significativa entre pacientes com e sem sarcopenia para autopercepção da deglutição, domínio limitação, escore total da ESV e possibilidade de disfagia. Houve diferença significativa entre pacientes com e sem relato de dispneia nos três protocolos, exceto o domínio emocional da ESV. Houve correlação positiva entre os protocolos.

**Conclusão:**

Nestes pacientes em CP houve autopercepção negativa da deglutição, autorreferência de sintomas vocais e possibilidade de disfagia com correlação positiva entre os três protocolos. Houve diferença significativa entre autopercepção da deglutição, sintomas vocais e possibilidade de disfagia com maior impacto em pacientes com sarcopenia e dispneia.

## INTRODUÇÃO

O Cuidado Paliativo (CP) é uma abordagem que visa a promoção da qualidade de vida de pacientes e seus familiares, através da avaliação precoce e controle de sintomas físicos, sociais, emocionais e espirituais desagradáveis no contexto de doenças que ameaçam a continuidade da vida^(1)^. A assistência é realizada por uma equipe multiprofissional durante o período do diagnóstico, adoecimento, finitude e luto, e visa apoio à família e ao paciente de acordo com suas preferências e necessidades^(1,2)^.

Neste contexto, o fonoaudiólogo desempenha papel fundamental no manejo da deglutição e da comunicação, guiado pelos princípios bioéticos de autonomia, beneficência, não maleficência e justiça. Sua atuação envolve a avaliação e intervenção na disfagia, a adaptação de estratégias comunicativas e o apoio à tomada de decisão compartilhada, respeitando as preferências e os desejos do paciente, com o propósito de aliviar o sofrimento, preservar a dignidade e manter a qualidade de vida até o fim^(3)^.

No Brasil, a estimativa de pacientes que precisavam de assistência de CP, em 2000, era de 662.065 e, para 2040, a estimativa mínima será de 1.166.279, demonstrando a cada dia o aumento da demanda para CP^(2)^.

Pacientes em CP frequentemente apresentam disfagia e disfonia devido às doenças neurodegenerativas ou neoplásicas que podem alterar a fisiologia da deglutição e fonação^(4)^. São comuns queixas relacionadas à deglutição nessa população^(5,6)^ e cerca de 70% dos pacientes são disfágicos para pelo menos uma consistência^(5)^.

Um outro estudo^(6)^ realizado em um serviço de CP revelou que 56% dos pacientes apresentavam disfonia com diminuição significativa do tempo máximo fonatório, além de alterações nos órgãos fonoarticulatórios e presença de disartrofonias. Esses resultados destacam a necessidade de uma avaliação detalhada da função vocal e fonoarticulatória em pacientes em CP, uma vez que essas alterações podem impactar significativamente a comunicação e a qualidade de vida desses indivíduos^(6)^. A identificação precoce desses problemas é crucial para o desenvolvimento de estratégias de intervenção que possam melhorar a função vocal e a capacidade de comunicação desses pacientes.

O rastreamento da possibilidade de disfagia é uma estratégia simples para minimizar o impacto da disfagia por intervenção precoce, além de colaborar com a qualidade de vida do paciente^(7)^. Em um estudo realizado em um hospital universitário, com uma amostra de 909 pacientes, foi encontrada possibilidade de disfagia em 10,5% e, dentro dos pacientes com risco, 4,2% apresentavam disfonia, que é um fator de risco para desenvolvimento da disfagia^(7)^.

Diante da prevalência dos sintomas de deglutição e de voz em pacientes em CP, é importante verificar a autopercepção relacionada à deglutição, aos sintomas vocais e à possibilidade de disfagia. Essa autoavaliação pode ser realizada por meio de instrumentos de autopercepção específicos, como estratégia para reforçar a intervenção precoce fonoaudiológica. Desta forma, cumpre-se o principal objetivo do CP: promover qualidade de vida ao indivíduo e oferecer subsídios para a tomada de decisão clínica. Portanto, este estudo teve como objetivo identificar, comparar e correlacionar a autopercepção da deglutição, de sintomas vocais e a possibilidade de ocorrência de disfagia nos pacientes em CP.

## MÉTODO

Trata-se de um estudo do tipo observacional, transversal e analítico, aprovado pelo Comitê de Ética e Pesquisa (CEP), da instituição de origem, sob o número 6.039.141. Todos os pacientes assinaram o Termo de Consentimento Livre e Esclarecido (TCLE).

A coleta de dados foi realizada por amostra de conveniência, entre abril de 2023 e maio de 2024. Foram incluídos pacientes internados em um hospital universitário, no setor de CP, com diferentes diagnósticos, categorizados conforme os graus de predominância dos cuidados paliativos, a saber: cuidados complementares, cuidados predominantes, cuidados exclusivos (totais) e fase de terminalidade/morte^(8,9)^, sem restrição de sexo, maiores de 18 anos. Foram excluídos pacientes sem condições clínicas e/ou interesse em responder os protocolos.

Os dados clínicos e sociodemográficos foram obtidos por meio da análise dos prontuários, na qual para verificação da possibilidade de sarcopenia, foi utilizado o instrumento de rastreamento SARC-F^(10)^. Os pacientes responderam a três protocolos, de forma autorrelatada, em um único momento:

1. O *Eating Assessment Tool* (EAT-10) é um instrumento de autopercepção da deglutição, utilizado em pacientes com diferentes doenças de base. Possui dez questões simples com possibilidade de pontuação de 0 a 4, no qual 0 não é um problema e 4 é um problema muito grande. Fornece informações sobre funcionalidade, impacto emocional e sintomas físicos, nos quais um problema de deglutição pode acarretar na vida de um indivíduo. O ponto de corte do instrumento é três, indicando autopercepção negativa e necessidade de avaliação fonoaudiológica formal^(11,12)^.
2. A Escala de Sintomas Vocais (ESV) é considerada a mais rigorosa e psicometricamente robusta para a autoavaliação vocal^(13)^, sobre a percepção de sintomas vocais e o quanto isso interfere na voz. São 30 itens divididos em três domínios: limitação (15 itens), emocional (8 itens) e físico (7 itens), pontuados de acordo com a frequência de ocorrência dos sintomas em: nunca (0), raramente (1), às vezes (2), quase sempre (3) e sempre (4). É realizada a somatória simples da pontuação, que pode variar de 0 a 120 pontos, em que quanto maior, pior a autopercepção de sintomas vocais o indivíduo apresenta, sendo sua nota de corte para diferenciação entre indivíduos vocalmente saudáveis e disfônicos de 16 pontos no escore total, 11,5 no domínio de limitação, 6,5 no físico e 1,5 no emocional^(13)^.
3. O Rastreamento de Disfagia Orofaríngea em Idosos no ambiente hospitalar (RaDI-H) é uma ferramenta de autoavaliação da disfagia em pacientes idosos. Apresenta nove questões de fácil aplicação, voltadas à coleta de informações de sintomas e queixas relacionadas à deglutição^(14,15)^. As possíveis respostas para cada questão são “não”, “sim” ou “não sabe”, e na presença de resposta positiva, investiga-se a frequência do sinal/sintoma por meio das opções “às vezes” ou “sempre”. Com as pontuações variando de 0 a 2 (“não” = zero; “sim” = 02; “às vezes” = 01; “sempre” = 02; “não sabe” = 02)^(14,15)^. O escore total varia de 0 a 18 pontos, e o ponto de corte para considerar positivo, com necessidade de encaminhamento para avaliação diagnóstica, é de 4 pontos^(14,15)^.

Na presença de autopercepção negativa da deglutição, de sintomas vocais e/ou possibilidade de disfagia, os pacientes foram encaminhados para a equipe de Fonoaudiologia para realização de avaliação fonoaudiológica formal e acompanhamento.

Para a análise e interpretação dos resultados, foram utilizados os programas Microsoft Excel e Jamovi, versão 2.0. Para comparar os escores do EAT-10, ESV e RaDI-H, foi utilizado o teste de Mann-Whitney, e para correlação entre os escores dos protocolos foi utilizado o teste de Correlação de Spearman, ambos considerando o nível de significância de 5% (p≤0,05). Os coeficientes de correlação foram classificados como: <0,3 (fraca), ≥ 0,3 a <0,6 (moderada), ≥ 0,6 a <0,9 (forte), ≥ 0,9 a <1,0 (muito forte) e r = 1,0 (perfeita)^(16)^.

## RESULTADOS

Participaram 50 pacientes, sendo 29 (58%) do sexo masculino. A maioria tinha mais de 60 anos (64%), com média de idade de 67 anos (±13,6). Quanto à doença de base, 33 (66%) pacientes foram diagnosticados com câncer. A via oral foi a principal forma de alimentação para 37 pacientes (74%). Houve possibilidade de sarcopenia em 23 (46%) pacientes e autorrelato de dispneia em 26 (52%).

Foi observada autopercepção negativa da deglutição em 40 pacientes (80%). Em relação aos sintomas vocais, 29 pacientes (58%) apresentaram escore total acima da nota de corte. No domínio limitação 22 pacientes (44%) apresentaram pontuação acima da nota de corte, seguido pelos domínios físico (n=16; 32%) e emocional (n=11; 22%). A possibilidade de disfagia foi observada em 31 pacientes (62%).

A pontuação média do EAT-10 foi de 10,5 (DP±10,4), com mediana de 7, sendo superior à nota de corte. Em relação à ESV, o domínio limitação apresentou média de 14,5 (DP±12) com mediana de 12, sendo acima da nota de corte. A média do domínio emocional foi 1,24 (DP±3), com mediana de 0 abaixo da nota de corte, e do domínio físico foi 6 (DP±5), com mediana de 5, ou seja, abaixo da nota de corte. A média do escore total da ESV foi 21,6 (DP±19), com mediana de 19,5, acima da nota de corte. O escore médio do RaDI-H foi de 5,44 (DP±3,8), com mediana de 4 superior a nota de corte (Tabela 1).

**Tabela 1:** Descrição dos dados do EAT 10, ESV e RaDI-H dos pacientes em Cuidados Paliativos (n=50)

Variáveis	EAT 10	ESV Limitação	ESV Emocional	ESV Físico	ESV Total	RaDI-H
Média (± DP)	10,5 (±10,4)	14,5 (± 12)	1,24 (± 3)	6 (± 5)	21,6 (± 19)	5,44 (± 3,8)
Mediana	7	12	0	5	19,5	4

**Legenda:** DP = Desvio padrão; EAT-10 = Eat Assessment Tool; ESV = Escala de Sintomas Vocais; RaDI-H = Rastreamento de Disfagia Orofaríngea em Idosos em Ambiente Hospitalar

Verificou-se diferença estatisticamente significativa entre indivíduos com e sem sarcopenia em relação à autopercepção da deglutição (p=0,005), ao domínio limitação da ESV (p=0,041), ao escore total da ESV (p=0,044), e à possibilidade de disfagia (p=0,007). Não houve diferença significativa no domínio físico (p=0,102) e emocional (p=0,765) da ESV, embora pacientes com sarcopenia tenham apresentado médias maiores (Tabela 2).

**Tabela 2:** Comparação dos dados do EAT 10, ESV e RaDI-H com dados clínicos dos pacientes em cuidados paliativos (n=50)

Variáveis		Sarcopenia			Dispneia		
		Sim	Não	p-valor	Sim	Não	p-valor
EAT-10		12	8,7	0,005*	15,4	6,2	0,001*
ESV	Limitação	19,8	8,6	0,041*	18,6	10,9	0,001*
	Emocional	1,5	0,9	0,765	1,2	1,2	0,233
	Físico	8,4	3,3	0,102	6,9	5	0,001*
	Total	29	12,9	0,044*	26,8	17	0,001*
RaDI-H		7	3,6	0,007*	6,9	4	0,001*

* p ≤ 0.05 = correlação significativa;

**Legenda**: EAT-10 = Eat Assessment Tool; RaDI-H = Rastreamento de Disfagia Orofaríngea em Idosos em Ambientes Hospitalares; ESV = Escala de Sintomas Vocais

Foram observadas diferenças significativas entre pacientes com e sem relato de dispneia (p=0,001) em todos os protocolos, exceto o domínio emocional da ESV, com médias iguais entre os pacientes (p=0,233) (Tabela 2).

Houve correlação positiva forte entre o EAT-10 e RaDI-H (r = 0,664) e o domínio físico da ESV (r = 0,653), correlação positiva moderada entre EAT-10 e o domínio limitação da ESV (r = 0,492) e o escore total (r = 0,556). O RaDI-H apresentou correlação positiva forte com o domínio limitação (r = 0,654), físico (r = 0,644) e escore total da ESV (r = 0,689). Além disso, observou-se correlação positiva fraca entre EAT-10 e o domínio emocional da ESV (r = 0,010), bem como entre RaDI-H e o domínio emocional da ESV (r = 0,246) (Tabela 3).

**Tabela 3:** Correlação dos dados do EAT 10, ESV e RaDI-H dos pacientes em cuidados paliativos (n=50)

Variáveis	EAT 10	RaDI-H
RaDI-H	0,664**	
ESV Limitação	0,492**	0,654**
ESV Emocional	0,010**	0,246**
ESV Físico	0,653**	0,644**
ESV Total	0,556**	0,689**

** Rho de Spearman = coeficiente de correlação;

**Legenda**: EAT-10 = Eat Assessment Tool; RaDI-H = Rastreamento de Disfagia Orofaríngea em Idosos em Ambientes Hospitalares; ESV = Escala de Sintomas Vocais

## DISCUSSÃO

O perfil de pacientes em CP varia segundo a literatura^(17)^, embora haja prevalência de pacientes do sexo masculino com mais de 60 anos, o que corrobora os resultados deste estudo. Acredita-se na associação de menor autocuidado em comparação às mulheres e maior propensão à prática de hábitos prejudiciais, como o consumo de substâncias ilícitas, álcool e tabaco^(17)^, os quais podem influenciar no aumento de comorbidades e complicações de saúde mais graves^(17)^.

Em relação à idade, pacientes mais idosos são frequentemente afetados por doenças degenerativas e complicações decorrentes do declínio fisiológico natural do envelhecimento, o que intensifica a necessidade de CP^(17)^. Em um estudo realizado em 2019^(17)^, foi observado que 54% dos pacientes eram do sexo masculino similar aos resultados deste estudo, porém com faixa etária entre 41 e 50 anos. Em outra pesquisa^(18)^, houve prevalência de mulheres, com média de 85 anos de idade entre os pacientes em CP. Essas divergências indicam que o perfil dos pacientes pode variar conforme o contexto e as características da população estudada.

A prevalência de câncer em CP se deve tanto pela elevada incidência quanto pela carga significativa de sintomas associados à doença e às sequelas do tratamento^(19,20)^. Segundo a literatura, nesses pacientes, há elevada autopercepção negativa da deglutição variando entre 58% e 77% em casos de câncer avançado^(19,20)^, com presença de sintomas vocais e possibilidade de disfagia^(21)^. Assim, torna-se crucial identificar a autopercepção da deglutição, o impacto de possíveis sintomas vocais e rastrear a possibilidade de disfagia nestes pacientes.

A alimentação por via oral (VO) tem benefícios clínicos e funcionais, como menor tempo de hospitalização, menor incidência de complicações clínicas e redução da sobrecarga do cuidador, além de envolver aspectos socioculturais e afetivos relevantes para a qualidade de vida^(22,23)^. Na presente pesquisa, a via oral foi a via predominante de alimentação, a qual é considerada menos invasiva, associada a melhor qualidade de vida e é, frequentemente, a via preferida pela família quando comparada aos métodos alternativos de alimentação^(23)^. No entanto, os achados deste estudo, assim como outros na literatura, demonstram que a presença de alimentação por VO total não exclui a possibilidade de disfagia, tampouco a autopercepção negativa da deglutição^(22-24)^.

A maioria dos pacientes (80%) apresentou autopercepção negativa da deglutição. A literatura corrobora esses achados, indicando que essa percepção está relacionada à piora da qualidade de vida em pacientes em CP^(6,25)^. Indivíduos em fase final da vida estão propensos a alterações na segurança e eficiência da deglutição, como resultado de múltiplos fatores, incluindo o impacto da doença de base e seus tratamentos, alterações estruturais, comprometimento sensorial e sensório-motor, fadiga, uso de medicações e alterações cognitivas^(25)^. Essa combinação de fatores contribui para a deterioração funcional da deglutição, influenciando diretamente a forma como o indivíduo percebe sua própria capacidade de deglutir^(6,25)^.

Em relação à autopercepção dos sintomas vocais, a maioria dos pacientes (58%) apresentou escores semelhantes aos de indivíduos disfônicos no escore total^(13)^, refletindo a autorreferência negativa da sua voz. Embora esse achado seja relevante, não foram encontrados estudos específicos na literatura que investiguem de forma direta a autopercepção vocal em pacientes em CP, o que destaca uma lacuna importante neste campo de pesquisa.

Segundo Gabriel et al.^(6)^ a disartrofonia e a redução do tempo máximo de fonação estão entre as principais alterações vocais observadas em pacientes em CP. Acredita-se que possa haver associação com a autopercepção vocal negativa nestes pacientes.

A maior parte da amostra (62%) apresentou possibilidade de disfagia, destacando a relevância do rastreamento fonoaudiológico nestes pacientes. Um estudo^(4)^ identificou que cerca de 30% dos pacientes em CP relataram, na triagem, queixas compatíveis com sinais sugestivos de penetração e/ou aspiração, sendo a xerostomia o sintoma mais frequente. Outro estudo^(26)^ revelou que tosse e engasgo são sintomas prevalentes. Quanto à influência na qualidade de vida, foi encontrada associação entre a presença da disfagia e o aumento da carga da doença, a qual pode levar ao surgimento de novos sintomas e impactar negativamente a qualidade de vida^(26)^.

Houve diferença estatisticamente significativa entre indivíduos com e sem sarcopenia para autopercepção da deglutição, domínio limitação e escore total da ESV, além da possibilidade de disfagia. Esses achados podem estar relacionados ao comprometimento da musculatura esquelética envolvida na deglutição e na produção vocal, causado pela sarcopenia^(24)^. A perda de força e coordenação muscular pode levar à autopercepção negativa da deglutição, aumento da possibilidade de disfagia e autopercepção de sintomas vocais^(24)^.

A degradação muscular, em pacientes em CP, decorre de fatores como desnutrição, inatividade física e inflamação crônica em doenças avançadas^(24)^, além de alterações hormonais e do uso de medicamentos como quimioterápicos e corticoides^(24)^. Condições como a caquexia aceleram ainda mais a perda de massa e função muscular, agravando o estado de fragilidade^(24)^. A literatura descreve maior prevalência de sarcopenia em pacientes oncológicos e do sexo masculino, perfil também identificado neste estudo. Em cuidados paliativos, a sarcopenia tem sido associada a menor tempo de sobrevida^(24)^.

Pacientes com autorrelato de dispneia tendem a apresentar escores mais elevados nos protocolos aplicados, o que reflete autopercepção negativa da deglutição, a presença de sintomas vocais e maior possibilidade de disfagia. Isso ocorre devido à dificuldade de coordenar adequadamente a respiração e deglutição, aumentando a ocorrência de penetração/aspiração^(26)^.

Fatores como redução da pressão subglótica, comprometimento glótico e interação entre sintomas respiratórios e musculares podem contribuir para o surgimento de sintomas vocais em pacientes em CP^(26)^. A presença de sarcopenia, fadiga, dispneia e alterações respiratórias compromete a coordenação pneumofonoarticulatória, podendo resultar em esforço fonatório compensatório e sobrecarga da laringe, ainda que de forma não voluntária ou exacerbada^(6,24,25,27,28)^. Essas alterações funcionais e estruturais da laringe impactam diretamente na qualidade vocal e na eficiência respiratória^(6,25)^. Nesse contexto, a interação entre os sistemas respiratório e laríngeo se torna crucial, pois sua desregulação pode prejudicar a qualidade vocal e afetar o processo de deglutição. Isso pode resultar no agravamento de sintomas como rouquidão, tosse, cansaço ao falar e dificuldades de deglutição, além de aumentar o risco de aspiração e complicações associadas. A compreensão dessa interação e de seus impactos é fundamental para a abordagem terapêutica eficaz desses pacientes.

A correlação positiva entre a autopercepção da deglutição, os sintomas vocais e a possibilidade de disfagia pode ser atribuída à sobreposição das estruturas anatômicas e musculares envolvidas nessas funções, como a laringe e a faringe^(6)^. Alterações nessas estruturas, comuns em condições neuromusculares e degenerativas, podem comprometer simultaneamente a deglutição e a voz, levando os pacientes a perceberem dificuldades em ambas^(6)^. A autopercepção negativa da deglutição e a presença de sintomas vocais frequentemente refletem alterações que também são identificadas no rastreamento da disfagia, reforçando a inter-relação entre essas variáveis no processo diagnóstico^(25)^.

Este estudo teve como limitação a coleta de dados em um único hospital, que não é referência em cuidados paliativos, com amostra restrita, o que impede a generalização dos resultados para outras populações ou contextos. A baixa rotatividade de pacientes e a presença de casos em processo ativo de morte ou delirium também dificultaram a aplicação dos protocolos. Para estudos futuros, recomenda-se a realização de pesquisas multicêntricas, envolvendo diferentes serviços de cuidados paliativos, para aumentar a representatividade da amostra e possibilitar a generalização dos achados. Além disso, é importante considerar a inclusão de hospitais de diferentes portes e especialidades, o que poderia fornecer uma visão mais abrangente das condições e necessidades dessa população.

## CONCLUSÃO

Os pacientes deste estudo, internados no setor de CP, apresentaram autopercepção negativa da deglutição, autorreferência de sintomas vocais e possibilidade de ocorrência de disfagia. Observou-se diferença estatisticamente significativa entre a autopercepção da deglutição, a presença de sintomas vocais e a possibilidade de ocorrência de disfagia, especialmente em pacientes com sarcopenia e dispneia. Além disso, foi identificada correlação positiva significativa entre os três protocolos avaliados, evidenciando uma inter-relação entre essas condições.
